# Reduction of the CD16^−^CD56^bright^ NK Cell Subset Precedes NK Cell Dysfunction in Prostate Cancer

**DOI:** 10.1371/journal.pone.0078049

**Published:** 2013-11-04

**Authors:** Kyo Chul Koo, Doo Hee Shim, Chang Mo Yang, Saet-Byul Lee, Shi Mun Kim, Tae Young Shin, Kwang Hyun Kim, Ho Geun Yoon, Koon Ho Rha, Jae Myun Lee, Sung Joon Hong

**Affiliations:** 1 Department of Urology and Urological Science Institute, Yonsei University College of Medicine, Seoul, Republic of Korea; 2 Department of Microbiology, Yonsei University College of Medicine, Seoul, Republic of Korea; 3 Brain Korea 21 Project for Medical Sciences, Yonsei University College of Medicine, Seoul, Republic of Korea; 4 ATgen, Sungnam, Republic of Korea; 5 Department of Biochemistry and Molecular Biology, Severance Medical Research Institute, Yonsei University College of Medicine, Seoul, Republic of Korea; Texas Tech University Health Sciences Center, United States of America

## Abstract

**Background:**

Natural cytotoxicity, mediated by natural killer (NK) cells plays an important role in the inhibition and elimination of malignant tumor cells. To investigate the immunoregulatory role of NK cells and their potential as diagnostic markers, NK cell activity (NKA) was analyzed in prostate cancer (PCa) patients with particular focus on NK cell subset distribution.

**Methods:**

Prospective data of NKA and NK cell subset distribution patterns were measured from 51 patients initially diagnosed with PCa and 54 healthy controls. NKA was represented by IFN-γ levels after stimulation of the peripheral blood with Promoca®. To determine the distribution of NK cell subsets, PBMCs were stained with fluorochrome-conjugated monoclonal antibodies. Then, CD16^+^CD56^dim^ and CD16^−^CD56^bright^ cells gated on CD56^+^CD3^−^ cells were analyzed using a flow-cytometer.

**Results:**

NKA and the proportion of CD56^bright^ cells were significantly lower in PCa patients compared to controls (430.9 pg/ml vs. 975.2 pg/ml and 2.3% vs. 3.8%, respectively; *p<0.001*). Both tended to gradually decrease according to cancer stage progression (*p* for trend = *0.001*). A significantly higher CD56^dim^-to-CD56^bright^ cell ratio was observed in PCa patients (41.8 vs. 30.3; *p<0.001*) along with a gradual increase according to cancer stage progression (*p* for trend = *0.001*), implying a significant reduction of CD56^bright^ cells in relation to the alteration of CD56^dim^ cells. The sensitivity and the specificity of NKA regarding PCa detection were 72% and 74%, respectively (best cut-off value at 530.9 pg/ml, AUC = 0.786).

**Conclusions:**

Reduction of CD56^bright^ cells may precede NK cell dysfunction, leading to impaired cytotoxicity against PCa cells. These observations may explain one of the mechanisms behind NK cell dysfunction observed in PCa microenvironment and lend support to the development of future cancer immunotherapeutic strategies.

## Introduction

Natural killer (NK) cells serve a major role in the innate and adaptive immune responses against tumor transformation or pathogen-infected cells [1]. NK cells exert natural cytotoxicity to eliminate malignant cells without prior sensitization or class I MHC restriction [1], [2]. Furthermore, NK cells stimulate the adaptive immune response by secreting proinflammatory cytokines to counteract the escape mechanisms promoted by tumor cells [3]. Progress has been made in understanding the biology of NK cells; nonetheless, further clarification remains regarding anti-tumor effects of NK cell activity (NKA) and patterns of subset distribution in PCa.

NK cells are defined phenotypically by their expression of CD56 and lack of CD3 expression [4]. According to membrane densities of CD56 and CD16, NK cells are classified into CD16^+^CD56^dim^ and CD16^−^CD56^bright^ subsets [5]. The majority are CD56^dim^ cells that mainly exert potent cytotoxicity [6]. In contrast, CD56^bright^ cells mediate low cytotoxicity but acquire greater cytolytic activity than CD56^dim^ cells upon activation due to release of proinflammatory cytokines such as IFN-γ [7]. The level of IFN-γ, i.e., NKA, is generally associated with oncological prognosis, which implies the essential role of differential NK cell subset expression in the immune regulation of tumor cells [8]. NKA has shown to serve an important role in surveillance and in the elimination of tumor cells [9]. Studies have shown that low NKA leads to high levels of tumor occurrence and metastasis, and that its degree correlates with invasiveness of malignancy [10]. On the contrary, high NKA has been shown to correlate with lower incidence of tumors, and their infiltration in certain tumors, i.e., melanoma, head and neck squamous cell carcinomas, is an indicator for a better oncological outcome [11], [12].

There is accumulating evidence that an impaired immune response is a crucial factor in the pathogenesis of prostate cancer (PCa) [13], [14]. NK cell dysfunction has been implicated in PCa along with a variety of tumors [15], [16]. Despite several proposed mechanisms including reduced number, immunosuppressive cytokines, and receptor repertoire imbalance, the pathophysiology of NK cell dysfunction in PCa is not fully understood [5]. Regarding the role of NKA in tumor suppression, harnessing the mechanisms of NK cells could clearly be an important component for successful immunotherapy against PCa. Prostate-specific antigen (PSA) is the most widely used serum marker that has revolutionized the early detection and management of PCa. However, the relative lack of cancer-specificity and lack of an upper or lower threshold value are major drawbacks.

To address these issues, NKA and the distributions of CD56^dim^ and CD56^bright^ subsets were analyzed between PCa patients and controls. Our findings indicate that immunoregulation in PCa is impaired due to a reduction in NKA preceded by redistribution of NK cell subsets. Moreover, evaluation of the diagnostic performance of NKA revealed that it may be applied as a supportive marker in addition to PSA.

## Materials and Methods

### 1. Patients and Controls

This prospective cross-sectional analysis involved 51 patients with newly diagnosed biopsy-proven PCa due to a PSA elevation noted on health examinations from March to December, 2012. 54 age-matched controls were self-volunteered healthy individuals whose prostate volume, PSA, and DRE were within normal accepted ranges. None of the patients had received prior treatment for PCa, were known to have immunological or other malignant conditions, and were all free of active infection or inflammation as assessed by white blood cell count <10,000 cells/µl and C-reactive protein <1.0 mg/L (Table 1). All controls were free from inflammatory conditions without prior exposure to immunosuppressive agents. Independent approval was obtained from Yonsei University Ethics Committee (4-2011-0660), with all blood samples collected after obtaining informed consent prior to radical prostatectomy. All participants provided written consent to participate in the current study.

**Table 1:** Demographic data of clinicopathological characteristics of PCa patients and controls.

	Patients	Controls	*p*-value
***Clinical characteristics***			
n	51	54	NS
Age (years)	63.7±0.98	61.6±0.78	*0.092*
BMI	24.4±0.44	24.1±0.35	*0.705*
Preoperative PSA (ng/ml)	16.9±3.5	1.1±0.15	*<0.001*
Prostate volume (gm)	34.2±1.6	28.8±1.8	*0.02*
WBC (cells/µl)	6463±236	6278±228	*0.874*
% Lymphocyte	31.4±1.1	33.2±0.9	*0.153*
% Neutrophil	57.9±1.4	55.6±1.3	*0.269*
C-reactive protein	<1.0	<1.0	NS
***Tumor characteristics***			
ECE	27 (53%)		NS
SV invasion	9 (16%)		NS
LN metastasis	8 (15%)		NS
TNM stage			NS
II	18 (36%)		
III	24 (47%)		
IV	9 (17%)		
Pathologic Gleason score			NS
6	10 (19%)		
7	25 (49%)		
8	8 (17%)		
9	8 (15%)		

All values are given as means ± SE.

NS = not significant; ECE = extracapsular extension; SV = seminal vesicle; LN = lymph node.

### 2. NK Cell Activity

Cytotoxic activity of NK cells was determined using the NK Vue-Kit® (ATgen, Sungnam, Korea). Whole blood was collected using BD Vacutainer® heparin^N1^ tubes. 1 ml of whole blood was incubated for 24 hrs, at 37°C, under 5% CO_2_ with indicated dose of Promoca® and 1 ml of RPMI 1640 media. Cell-free supernatants were harvested, and IFN-γ levels were determined according to manufacturer’s protocols.

### 3. NK Cell Subset Distribution

#### 3.1. Preparation of PBMCs

3 ml of heparinized venous blood was obtained and analyzed within 4 h of collection. PBMCs were isolated by density gradient centrifugation using CPT® cell preparation tubes (BD Vacutainer®) at 1600 g for 20 min at 20°C. The collected PBMCs (1−2×10^6^ cells/ml) were washed and resuspended in 5% fetal bovine serum (FBS)+phosphate buffered saline (PBS).

#### 3.2. Antibody staining

For the expression of CD3, CD16, and CD56 on NK cells, PBMCs were stained with Alexa-anti-CD3, PE-anti-CD16, and FITC-anti-CD56 fluorochrome-conjugated monoclonal antibodies (BD Biosciences). After staining for 30 min at 4°C, cells were washed extensively and fixed in 1% paraformaldehyde-PBS until assessment.

#### 3.3. Flow cytometry

To determine the total percentage of NK cells gated in the CD3^−^CD56^+^ cell population, at least 10,000 target cells were acquired by LSRII flow-cytometry (BD Biosciences). The distribution of CD16^+^CD56^dim^ NK cells and CD16^−^CD56^bright^ NK cells gated from the CD3^−^CD56^+^ cell population is presented as the percentage of total NK cells. For each sample, the data were further analyzed by FlowJo 8.1.1.1 (Tree Star, Inc., Ashland, OR, USA).

### 4. Cancer Stage Classification

PCa staging was determined according to the 7^th^ American Joint Committee on Cancer (AJCC) TNM system. Stage distribution and pathological characteristics are shown in Table 2. Pathology was confirmed by a single pathologist.

**Table 2:** Comparisons of NK cell activity, % total NK cell population, distribution of CD56^dim^ and CD56^bright^ subsets, and the CD56^dim^-to-CD56^bright^ ratio between patients and controls.

	Patients				Controls	*p*
	Total	Stage II	Stage III	Stage IV		
NK cell activity (pg/ml)	430.9±67.1	546.1±136.8	427.8±87.9	194.5±73.8	975.2±85.7	*<0.001*
NK cell population						
cells/µl	3865±1944	3552±1351	3615±1462	4194±1762	4662±1826	*0.312*
%†	20.2±1.6	16.9±1.8	21.8±1.9	23.9±5.8	21.8±1.7	*0.595*
CD56^dim^ subset						
cells/µl	3258±1837	2959±935	3023±1659	3774±1769	4298±1134	*0.208*
%‡	85.36±1.2	83.5±1.8	86.2±1.9	88.1±3.2	88.9±0.8	*0.103*
CD56^bright^ subset						
cells/µl	96.5±61.3	94.2±30.3	77.5±40.8	84.5±19.3	202.5±90.1	*<0.001*
%‡	2.3±0.2	2.7±0.3	2.2±0.2	1.7±0.4	3.8±0.3	*<0.001*
CD56^dim^-to-CD56^bright^ ratio	41.8±2.3	35.8±3.3	43.6±3.3	54.4±7.4	30.3±3.1	*<0.001*

† Expressed as a percentage of total PBMCs.

‡ Expressed as a percentage of total NK cells.

Patients were further grouped according to cancer stage. All data represented as mean ± SE.

### 5. Statistical Analysis

Statistical analyses were performed using Mann-Whitney U tests when comparing unpaired two-group data and Kruskal-Wallis tests with Bonferroni post-hoc correction when comparing more than two groups. The accuracy of NKA and the CD56^dim^-to-CD56^bright^ ratio in detecting PCa was determined by receiver operating characteristics-derived area under the curve (AUC). Correlation analysis was used to evaluate associations among NKA, CD56^dim^-to-CD56^bright^ ratio, and clinicopathological variables. Statistical analyses were performed using SPSS (v.18.0).

## Results

### 1. Demographic Data

All patients and controls were clinically and pathologically investigated with respect to factors shown in Table 1. Factors that may influence one’s immune status manifested no differences between groups.

### 2. Frequency of NK Cells and Distribution of CD56^dim^ and CD56^bright^ NK Cell Subsets

**Figure 1:**
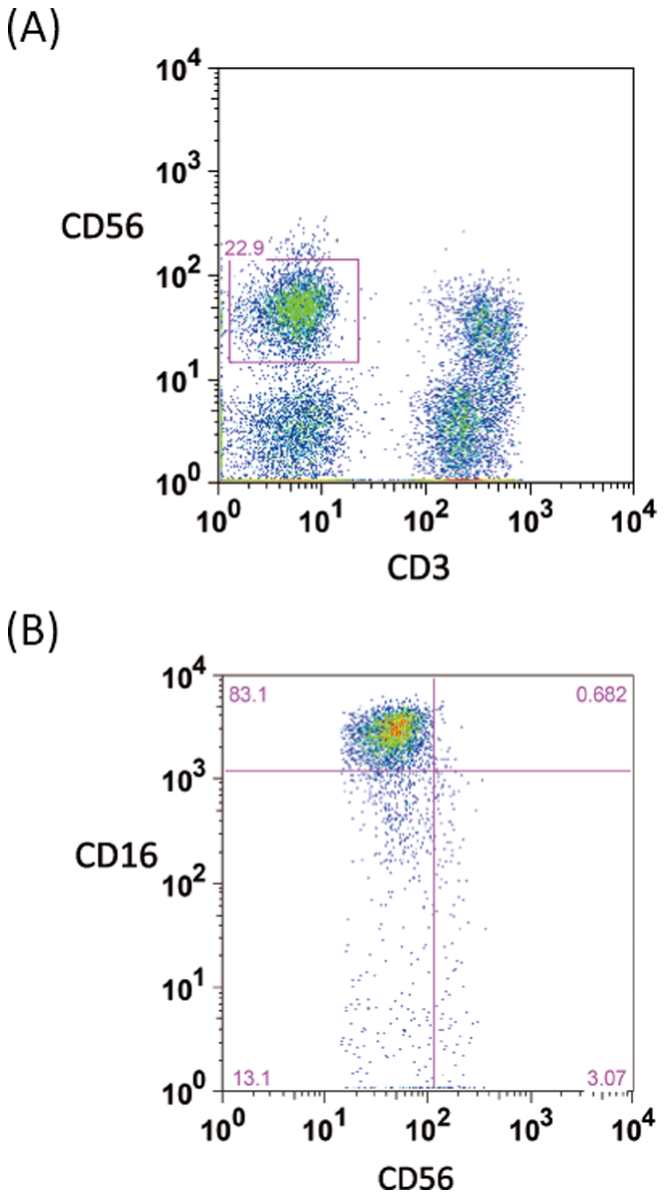
(A) Representative flow cytometric data of the distribution of total NK cell population represented by CD3^−^CD56^+^ cells. (B) Representative flow cytometric data of two major NK cell subsets detected in peripheral blood. Left, upper box: CD16^+^CD56^dim^ NK cell subset. Right, lower box: CD16^−^CD56^bright^ NK cell subset.

**Figure 2:**
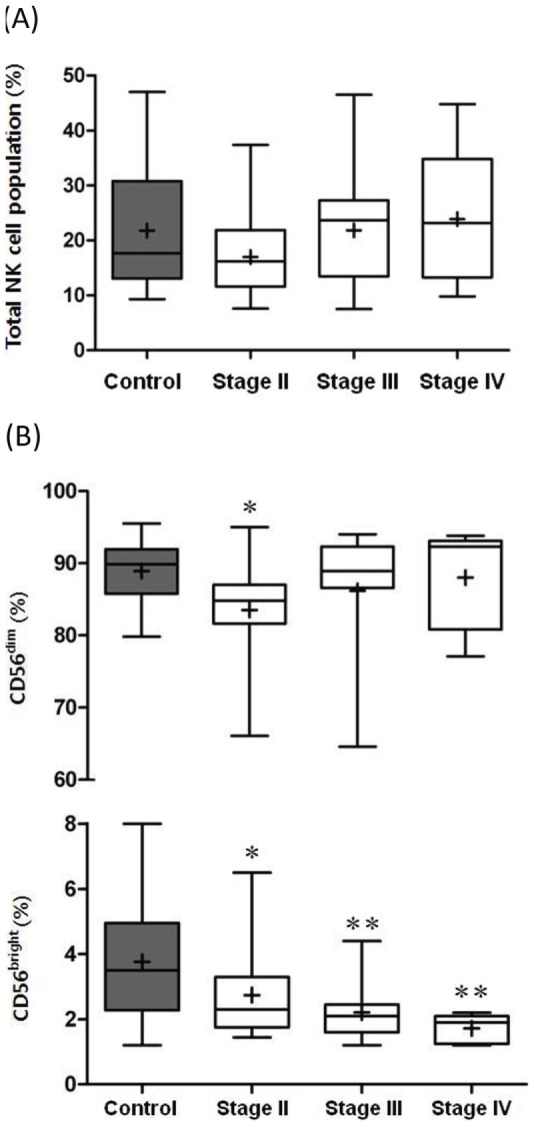
(A) Boxplot diagrams showing flow cytometric distribution results of total NK cell population % between controls and patients, grouped according to cancer stage. No significant differences of total NK population were noted between patients and controls, and within stage groups. (B) Boxplot diagrams showing flow cytometric distribution results of CD56^dim^ and CD56^bright^ subset distributions within total NK cells between controls and patients, grouped according to cancer stage. *p<0.05, **p<0.01 in relation to controls.

Representative flow cytometric data shows the distribution of total NK cell population represented as CD3^−^CD56^+^ cells (Fig. 1A) and two major subsets, CD16^+^CD56^dim^ and CD16^−^CD56^bright^, expressed as a percentage of total NK cells (Fig. 1B). Total NK circulating frequencies did not differ between patients and controls or between cancer stage groups (Fig. 2A; Table 2). However, a preferential decrease in frequency of CD56^bright^ cells was noted in patients. Moreover, CD56^bright^ cells tended to gradually decrease according to cancer stage progression, i.e., extracapsular extension, LN or adjacent organ metastasis (Fig. 2B; Table 2). A significantly higher CD56^dim^-to-CD56^bright^ NK cell ratio was observed in patients compared to controls, with a tendency to increase according to stage progression (*p* for trend = *0.001*) (Table 2).

### 3. NK Cell Activity

**Figure 3:**
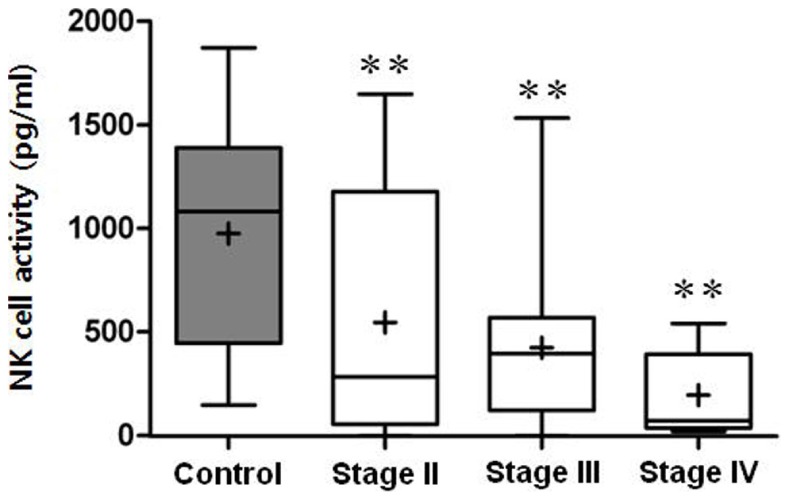
Boxplot diagram comparing NK cell activity between controls and patients grouped according to cancer stage. **p<0.01 in relation to controls.

Results obtained are presented in Fig. 3 and Table 2. As indicated, patients showed significantly lower NKA. According to stage progression, those with higher stages showed a greater reduction of NKA (*p* for trend <*0.001*).

### 4. Analysis by ROC Curves

**Figure 4:**
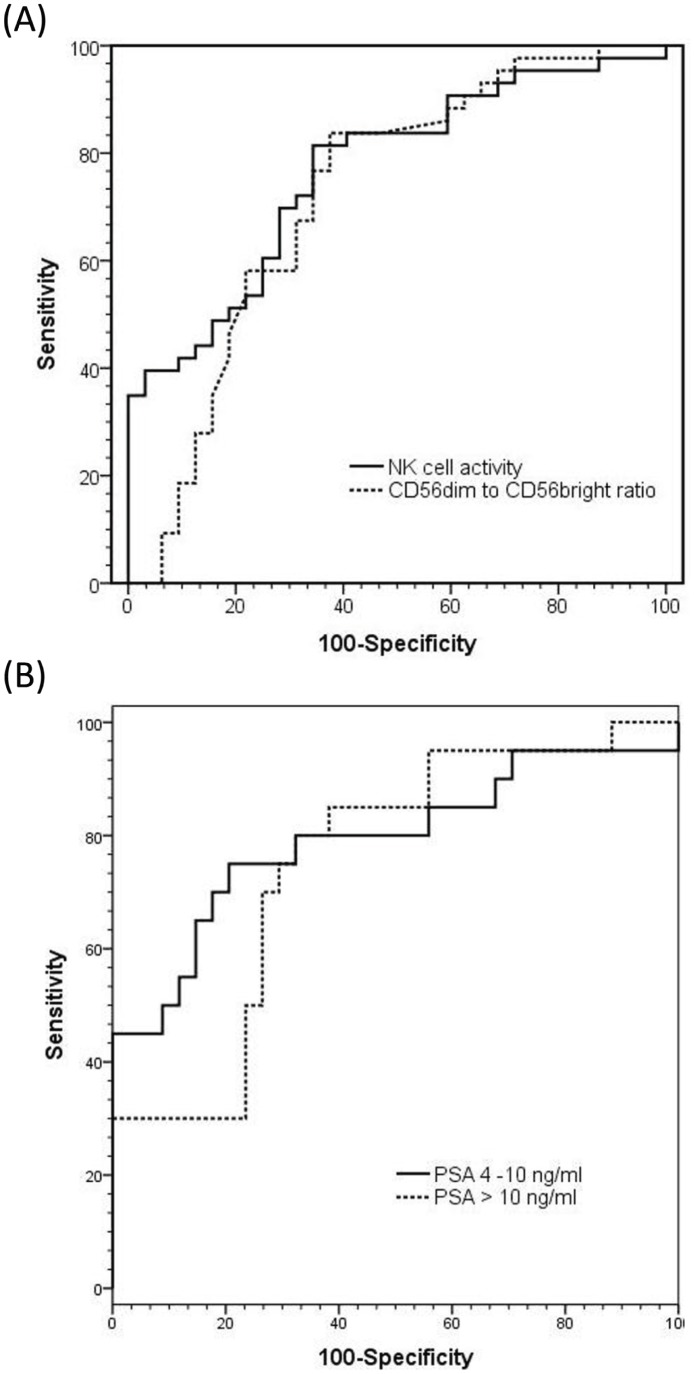
(A) ROC curves comparing the performances of NK cell activity and CD56^dim^-to-CD56^bright^ ratio measurements. (AUC = Area Under the Curve). (B) ROC curves comparing the performances of NK cell activity measurement according to PSA grouped as; 4 to 10 ng/ml and greater than 10 ng/ml. (AUC = Area Under the Curve).

ROC curves and best cut-off values were used to calculate the sensitivity and specificity of NK cell-related parameters (Table 3). The sensitivity and specificity of NKA with respect to PCa detection were 72% and 74%, respectively, whereas the CD56^dim^-to-CD56^bright^ cell ratio showed a sensitivity of 66% and a specificity of 71% (Fig. 4A). In further analysis, the sensitivity and specificity of NKA were determined according to two PSA values grouped as 4 to 10 ng/ml, which is the diagnostic grey-zone, and levels greater than 10 ng/ml. At a set specificity of 74%, NKA for PSA values within the grey-zone showed higher sensitivity (73% vs. 70%) and AUC (0.82±0.06 vs. 0.76±0.07) relative to PSA values greater than 10 ng/ml (Fig. 4B).

**Table 3:** Comparisons of sensitivity and specificity of NK cell activity and CD56^dim^-to-CD56^bright^ ratio to detect PCa.

Diagnostic test	AUC	Sensitivity (95% CI)	Specificity (95% CI)	Cut-off value
NK cell activity	0.79±0.05	72%	74%	530.9 pg/ml
PSA 4–10 ng/ml	0.82±0.06	73%	74%	
PSA >10 ng/ml	0.76±0.07	70%	74%	
CD56^dim^-to-CD56^bright^ ratio	0.72±0.06	66%	71%	35.5

AUC = area under the curve.

The sensitivity of NK cell activity for corresponding PSA range is set at 74% specificity.

### 5. NK Cell Activity and CD56^dim^-to-CD56^bright^ Cell Ratio According to Clinicopathological Variables

NKA showed negative correlations with PSA, cancer stage, and the CD56^dim^-to-CD56^bright^ ratio. On the other hand, CD56^dim^-to-CD56^bright^ cell ratio showed positive correlations with PSA and cancer stage (Table 4). NKA and CD56^dim^-to-CD56^bright^ ratio was compared between controls and patients grouped according to clinicopathological variables (Table 5). Although CD56^dim^-to-CD56^bright^ ratio failed to discriminate patients with Gleason scores <7 and those without extracapsular extension from controls, all other subgroups were distinguishable from controls by NKA and CD56^dim^-to-CD56^bright^ ratio. Analysis in-between patient subgroups revealed significantly higher CD56^dim^-to-CD56^bright^ ratio in patients with pathologically confirmed LN metastasis (*p = 0.043*; data not shown).

**Table 4:** Correlation of NK cell activity and CD56^dim^-to-CD56^bright^ ratio between clinicopathological variables.

Variable	NK cell activity		CD56^dim^ to CD56^bright^ ratio	
	r	*p*	r	*p*
NK cell activity	NS	*NS*	−0.28	*0.01*
PSA	−0.41	*<0.001*	0.44	*<0.001*
Stage	−0.51	*<0.001*	0.44	*<0.001*
ECE	−0.12	*0.43*	0.28	*0.06*
SV invasion	−0.26	*0.08*	0.24	*0.11*
LN metastasis	−0.18	*0.24*	0.27	*0.08*
Gleason score	−0.08	*0.58*	0.14	*0.36*
Tumor volume	0.01	*0.99*	0.07	*0.67*
Prostate volume†	−0.22	*0.06*	0.13	*0.26*
Age†	−0.18	*0.12*	0.19	*0.09*
BMI†	−0.19	*0.08*	0.24	*0.03*

NS = not significant; ECE = extracapsular extension; SV = seminal vesicle; LN = lymph node.

All datasets analyzed by Spearman’s correlation analysis.

† Normal distributional variables were calculated by Pearson’s correlation analysis.

**Table 5:** Comparisons of NK activity and the CD56^dim^-to-CD56^bright^ ratio between controls and patients grouped according to clinicopathological variables.

Variable	n	Mean ± S.D		*P* *	
		NK activity (pg/ml)	ratio	NK activity (pg/ml)	ratio
Controls	54	975.2±85.7	30.3±3.1		
Patients					
PSA (ng/ml)					
4–10	27	373.1±99.8	38.9±2.5	*<0.001*	*0.007*
≥10	24	498.3±95.5	45.9±4.4	*0.002*	*0.004*
Gleason score					
<7	10	540.1±88.2	34.1±3.6	*0.041*	*0.174*
≥7	41	411.9±92.3	43.8±2.7	*<0.001*	*<0.001*
SV invasion					
(−)	42	487.4±87.5	39.8±3.2	*<0.001*	*0.002*
(+)	9	166.5±92.3	54.5±4.5	*<0.001*	*0.019*
ECE					
(−)	24	545.8±95.4	35.7±3.4	*0.004*	*0.097*
(+)	27	365.3±87.3	46.4±2.6	*<0.001*	*<0.001*
LN metastasis					
(−)	43	478.1±82.4	40.2±3.8	*0.001*	*0.003*
(+)	8	217.3±89.6	50.9±4.1	*<0.001*	*0.011*

SV = seminal vesicle; ECE = extracapsular extension; LN = lymph node.

* *P*-value for difference between controls and patient subgroup.

## Discussion

The aim of the present study was to clarify the role of NK cells in the immune response against PCa. Several mechanisms of PCa development and progression have been proposed, including hormonal, metabolic alterations, and immune response [6], [17]. There is accumulating evidence that different lymphocyte populations are involved in cell-mediated immunosuppression that leads to occurrence and progression of PCa [13], [18], [19]. However, there is limited information regarding the functional role of NK cells in the immune response to PCa. To address this issue, we investigated NKA as a marker for IFN-γ levels and the distribution of NK cell subsets in PCa patients. The results of our study indicate that impaired NKA is presumably preceded by a reduction in CD56^bright^ cells, and that the level of NKA could be utilized as a supportive diagnostic marker for PSA.

### 1. Preferential Reduction of CD56^bright^ NK Cells in PCa Patients

NK cells are functionally classified into CD56^dim^ and CD56^bright^ subsets. CD16^+^CD56^dim^ cells are effector cells with high quantities of cytolytic granules that express potent cytotoxicity against tumor cells [4]. CD16^−^CD56^bright^ cells release proinflammatory cytokines such as IFN-γ which drives inflammatory mechanisms that regulate tumor initiation, immunoevasion, survival, and outgrowth [20], [21]. Recent discoveries have revealed that CD56^bright^ cells constitute the majority of NK cells in lymphoid tissues and that they are not just a minor subpopulation among NK cells but are immature precursors of CD56^dim^ cells [22]. This work focuses on this particular subset, regarding its importance in the regulation of NK cell-mediated response against tumor cells.

Investigation on distributional patterns of NK cell subsets revealed a significant decrease of CD56^bright^ cells without alteration of CD56^dim^ cells. Previous studies on various tumor-bearing hosts have reported rather distinct interrelationships between CD56^dim^ and CD56^bright^ subsets. In contrast to our results, a reduction in CD56^dim^ cells without alteration of CD56^bright^ cells was noted in gastric and esophageal cancers [23]. On the other hand, a reduction in CD56^bright^ cells was observed in breast, head and neck cancers, and an equal distribution of CD56^dim^ cells in PCa; results that are consistent with the present study [7], [17].

### 2. Alteration of CD56^bright^ NK Cells as a Response Mechanism to Tumor Microenvironment

Our study primarily observed a preferential reduction of CD56^bright^ cells without alteration of CD56^dim^ cells. Although the underlying cause has not yet been clearly defined, two possible explanations can be raised; maturation process and recruitment process.

As mentioned, CD56^bright^ cells are accepted as precursors to CD56^dim^ cells, with each subset representing a distinct maturation stage [24]. Possibly, an excessive demand for effector cells in response to tumor may have provoked a transition of immature CD56^bright^ cells into CD56^dim^ cells. A similar explanation has been proposed for the reduction of CD56^bright^ cells in patients with head and neck cancers [7]. However, this presupposes a concomitant increase of CD56^dim^ cells, which was not observed in the present study.

An alternative explanation without demonstration is that peripheral CD56^bright^ cells may have been recruited to lymphoid tissue sites as metastatic LNs to acquire cytotoxicity. This idea was based on previous observations that CD56^bright^ cells preferentially accumulate in the T cell area of LNs until being activated to produce proinflammatory cytokines [7], [22,22]. Moreover, the observation that CD56^bright^ cells isolated from human LNs become strongly cytotoxic upon stimulation by IL-2 suggests that NK cells recruited to LNs might represent an immature pool of effector cells [25]. A significantly higher CD56^dim^-to-CD56^bright^ cell ratio in patients with pathologically confirmed LN metastasis was observed in our study, implying that these circulating cells may have been recruited to pathologic or secondary LNs in response to tumor. This is of relevance because LNs are usually the primary metastatic sites and CD56^bright^ cells are the primary subset found in LNs that counteract the metastatic cells [26]. To confirm this issue, it would be interesting to examine whether CD56^bright^ cells are accumulated in metastatic LNs following LN dissection.

### 3. NK Cell Dysfunction as a Consequence of Reduction of CD56^bright^ NK Cells

NKA was investigated to determine the influence of reduction of CD56^bright^ cells on cytolytic activity against tumor cells. Parallel to observations with CD56^bright^ cells, NKA was observed to be lower in PCa patients, along with a tendency to gradually decrease according to cancer stage progression. These findings are consistent with previous reports that showed NKA is compromised in a broad spectrum of hematological and solid tumors [8], [10], [27]. Several mechanisms of compromised NKA have been proposed, such as decreased number of tumor-infiltrating NK cells [23], increased surface receptors for immune suppressor factors [28], and inactivation of effector cells [10].

Correlations observed between NKA and CD56^dim^-to-CD56^bright^ cell ratio may be of direct relevance to suggest an additional mechanism that weak NKA is a consequence of reduced CD56^bright^ cells. CD56^bright^ cells are known to be major sources for IFN-γ [22], as observed in *in vitro* studies where CD56^bright^ cells were shown to preferentially proliferate in co-culture with immature dendritic cells and lipopolysaccharides to produce IFN-γ [29]. Also, stimulation of CD56^bright^ cells with transduced carcinoma cells resulted in an enhanced ability to produce IFN-γ and impart high cytotoxicity [6]. Further, *in vivo* studies have shown that tonsillar CD56^bright^ cells produce IFN-γ before maturation into effector cells [25]. Conversely, a reduction of CD56^bright^ cells was observed to induce impaired secretion of IFN-γ in patients with allergic rhinitis [30]. Considering these supportive findings that secretion of IFN-γ directly depends on CD56^bright^ cells, we suggest that the reduction of CD56^bright^ cells is a potential mechanism involved in low NKA, which leads to impaired cytotoxicity against PCa cells.

### 4. NK Cell Activity, a Supportive Diagnostic Marker for PSA

ROC curves revealed that NKA may serve as a supportive marker for PSA in diagnosing PCa. Although it is clear that PSA provides the highest diagnostic value for PCa, a major limitation is its lack of cancer-specificity which causes unnecessary risks and costs, especially in the diagnostic grey-zone [31]. Although ongoing challenges strive to develop novel methods of PCa detection, none have clearly outweighed the benefits against drawbacks [14]. This investigation raises the possibility that NKA may be utilized in combination with PSA to provide additional diagnostic value, especially for those within the diagnostic grey-zone.

This study was based on controls versus patients diagnosed with PCa due to elevated PSA on a routine health examination. Therefore, the absence of PCa patients with normal PSA (<4 ng/ml) was the major limitation of this study, which hampered a direct comparison of diagnostic yield between PSA and NKA. An extended population study is needed to confirm our preliminary findings and to assess cost-effectiveness.

### Conclusions

This observational study provided novel findings that CD56^bright^ cells serve an important role in adaptive response against PCa cells. This notion lends further support that longitudinal studies regarding NK cell immunosurveillance clearly deserve additional research to potentially lead to novel immunotherapeutic strategies for enhancing oncological outcomes of PCa.

## References

[1] Vivier E, Raulet DH, Moretta A, Caligiuri MA (2011) Innate or Adaptive Immunity? The Example of Natural Killer Cells. Science 331: 44–49. 21212348 10.1126/science.1198687 PMC3089969

[2] Koch J, Steinle A, Watzl C, Mandelboim O (2013) Activating natural cytotoxicity receptors of natural killer cells in cancer and infection. Trends Immunol 34: 182–191. 23414611 10.1016/j.it.2013.01.003

[3] Takahashi E, Kuranaga N, Satoh K, Habu Y, Shinomiya N, et al (2007) Induction of CD16+ CD56bright NK cells with antitumour cytotoxicity not only from CD16- CD56bright NK Cells but also from CD16- CD56dim NK cells. Scand J Immunol 65: 126–138. 17257217 10.1111/j.1365-3083.2006.01883.x

[4] Cooper MA, Fehniger TA, Caligiuri MA (2001) The biology of human natural killer-cell subsets. Trends Immunol 22: 633–640. 11698225 10.1016/s1471-4906(01)02060-9

[5] Watanabe M, Kono K, Kawaguchi Y, Mizukami Y, Mimura K, et al (2010) NK cell dysfunction with down-regulated CD16 and up-regulated CD56 molecules in patients with esophageal squamous cell carcinoma. Dis Esophagus 23: 675–681. 20545975 10.1111/j.1442-2050.2010.01073.x

[6] Dowell AC, Oldham KA, Bhatt RI, Lee SP, Searle PF (2012) Long-term proliferation of functional human NK cells, with conversion of CD56dim NK cells to a CD56bright phenotype, induced by carcinoma cells co-expressing 4-1BBL and IL-12. Cancer Immunol Immunother 61: 615–628. 22021067 10.1007/s00262-011-1122-3 PMC11029033

[7] Bauernhofer T, Kuss I, Henderson B, Baum AS, Whiteside TL (2003) Preferential apoptosis of CD56dim natural killer cell subset in patients with cancer. Eur J Immunol 33: 119–24. 12594840 10.1002/immu.200390014

[8] Lin CT, Yu MT, Li C, Ho YC, Shen CH, et al (2010) Dysfunction of natural killer cells in patients with transitional cell carcinoma. Cancer Lett 291: 39–45. 19931980 10.1016/j.canlet.2009.09.019

[9] Kim S, Iizuka K, Aguila HL, Weissman IL, Yokoyama WM (2000) In vivo natural killer cell activities revealed by natural killer cell-deficient mice. Proc Natl Acad Sci U S A 97: 2731–2736. 10694580 10.1073/pnas.050588297 PMC15998

[10] Takeuchi H, Maehara Y, Tokunaga E, Koga T, Kakeji Y, et al (2001) Prognostic significance of natural killer cell activity in patients with gastric carcinoma: a multivariate analysis. Am J Gastroenterol 96: 574–578. 11232710 10.1111/j.1572-0241.2001.03535.x

[11] Schleypen JS, Baur N, Kammerer R, Nelson PJ, Rohrmann K, et al (2006) Cytotoxic markers and frequency predict functional capacity of natural killer cells infiltrating renal cell carcinoma. Clin Cancer Res 12: 718–725. 16467081 10.1158/1078-0432.CCR-05-0857

[12] Vivier E, Ugolini S, Blaise D, Chabannon C, Brossay L (2012) Targeting natural killer cells and natural killer T cells in cancer. Nat Rev Immunol 12: 239–252. 22437937 10.1038/nri3174 PMC5161343

[13] Klein EA, Silverman R (2008) Inflammation, infection, and prostate cancer. Curr Opin Urol 18: 315–319. 18382242 10.1097/MOU.0b013e3282f9b3b7

[14] Basch E, Oliver TK, Vickers A, Thompson I, Kantoff P, et al (2012) Screening for prostate cancer with prostate-specific antigen testing: American Society of Clinical Oncology Provisional Clinical Opinion. J Clin Oncol 30: 3020–3025. 22802323 10.1200/JCO.2012.43.3441 PMC3776923

[15] Oikawa T, Kawai K, Ishiwata I, Ohno T, Akaza H (2003) Induction of potent antitumour natural-killer cells from peripheral blood of patients with advanced prostate cancer. BJU Int 92: 1009–1015. 14632866 10.1111/j.1464-410x.2003.04509.x

[16] Kastelan M, Kraljic I, Tarle M (1992) NK cell activity in treated prostate cancer patients as a probe for circulating tumor cells: hormone regulatory effects in vivo. Prostate 21: 111–120. 1384013 10.1002/pros.2990210204

[17] Sotosek S, Sotosek Tokmadzic V, Mrakovcic-Sutic I, Thomas MI, Dominovic M, et al (2011) Comparative study of frequency of different lymphocytes subpopulation in peripheral blood of patients with prostate cancer and benign prostatic hyperplasia. Wien Klin Wochenschr 123: 718–725. 22105113 10.1007/s00508-011-0096-7

[18] Ebelt K, Babaryka G, Figel AM, Pohla H, Buchner A, et al (2008) Dominance of CD4+ lymphocytic infiltrates with disturbed effector cell characteristics in the tumor microenvironment of prostate carcinoma. Prostate 68: 1–10. 17948280 10.1002/pros.20661

[19] Wagenlehner FM, Elkahwaji JE, Algaba F, Bjerklund-Johansen T, Naber KG (2007) Hartung R, Weidner W. The role of inflammation and infection in the pathogenesis of prostate carcinoma. BJU Int 100: 733–737. 17662075 10.1111/j.1464-410X.2007.07091.x

[20] Harlin H, Hanson M, Johansson CC, Sakurai D, Poschke I, et al (2007) The CD16- CD56bright NK cell subset is resistant to reactive oxygen species produced by activated granulocytes and has higher antioxidative capacity than the CD16+ CD56dim subset. J Immunol 179: 4513–4519. 17878347 10.4049/jimmunol.179.7.4513

[21] Zaidi MR, Merlino G (2011) The two faces of interferon-gamma in cancer. Clin Cancer Res 17: 6118–6124. 21705455 10.1158/1078-0432.CCR-11-0482 PMC3186825

[22] Poli A, Michel T, Theresine M, Andres E, Hentges F, et al (2009) CD56bright natural killer (NK) cells: an important NK cell subset. Immunology 126: 458–465. 19278419 10.1111/j.1365-2567.2008.03027.x PMC2673358

[23] Izawa S, Kono K, Mimura K, Kawaguchi Y, Watanabe M, et al. H2O2 production within tumor microenvironment inversely correlated with infiltration of CD56dim NK cells in gastric and esophageal cancer: possible mechanisms of NK cell dysfunction. Cancer Immunol Immunother 60: 1801–1810. 10.1007/s00262-011-1082-7 PMC11028881 21811786

[24] Chan A, Hong DL, Atzberger A, Kollnberger S, Filer AD, et al (2007) CD56bright human NK cells differentiate into CD56dim cells: role of contact with peripheral fibroblasts. J Immunol 179: 89–94. 17579025 10.4049/jimmunol.179.1.89

[25] Ferlazzo G, Munz C (2004) NK cell compartments and their activation by dendritic cells. J Immunol 172: 1333–1339. 14734707 10.4049/jimmunol.172.3.1333

[26] Sheikhi A, Saadati K, Salmani R, Yahaghi N, Siemens DR (2011) In vitro modulation of natural killer activity of human peripheral blood mononuclear cells against prostate tumor cell line. Immunopharmacol Immunotoxicol 33: 700–708. 21425925 10.3109/08923973.2011.561437

[27] Rabinovich GA, Gabrilovich D, Sotomayor EM (2007) Immunosuppressive strategies that are mediated by tumor cells. Annu Rev Immunol 25: 267–296. 17134371 10.1146/annurev.immunol.25.022106.141609 PMC2895922

[28] Yamaguchi Y, Takashima I, Funakoshi M, Kawami H, Toge T (1994) Defective natural killer activity in gastric cancer patients: possible involvement of suppressor factor receptor. In Vivo 8: 279–283. 7803704

[29] Vitale M, Della Chiesa M, Carlomagno S, Romagnani C, Thiel A, et al (2004) The small subset of CD56brightCD16- natural killer cells is selectively responsible for both cell proliferation and interferon-gamma production upon interaction with dendritic cells. Eur J Immunol 34: 1715–1722. 15162442 10.1002/eji.200425100

[30] Scordamaglia F, Balsamo M, Scordamaglia A, Moretta A, Mingari MC, et al (2008) Perturbations of natural killer cell regulatory functions in respiratory allergic diseases. J Allergy Clin Immunol 121: 479–485. 18061653 10.1016/j.jaci.2007.09.047

[31] Schroder FH (2012) Landmarks in prostate cancer screening. BJU Int 110 Suppl 13–7. 23046034 10.1111/j.1464-410X.2012.011428.x

